# A Latent Variable Partial Least Squares Path Modeling Approach to Regional Association and Polygenic Effect with Applications to a Human Obesity Study

**DOI:** 10.1371/journal.pone.0031927

**Published:** 2012-02-27

**Authors:** Fuzhong Xue, Shengxu Li, Jian'an Luan, Zhongshang Yuan, Robert N. Luben, Kay-Tee Khaw, Nicholas J. Wareham, Ruth J. F. Loos, Jing Hua Zhao

**Affiliations:** 1 Department of Epidemiology and Health Statistics, School of Public Health, Shandong University, Jinan, China; 2 MRC Epidemiology Unit and Institute of Metabolic Science, Cambridge, United Kingdom; 3 Department of Epidemiology, School of Public Health and Tropical Medicine, Tulane University, New Orleans, Louisiana, United States of America; 4 Strangeways Research Laboratory, Department of Public Health and Primary Care, University of Cambridge, Cambridge, United Kingdom; 5 Clinical Gerontology Unit, School of Clinical Medicine, University of Cambridge, Cambridge, United Kingdom; University of Utah, United States of America

## Abstract

Genetic association studies are now routinely used to identify single nucleotide polymorphisms (SNPs) linked with human diseases or traits through single SNP-single trait tests. Here we introduced partial least squares path modeling (PLSPM) for association between single or multiple SNPs and a latent trait that can involve single or multiple correlated measurement(s). Furthermore, the framework naturally provides estimators of polygenic effect by appropriately weighting trait-attributing alleles. We conducted computer simulations to assess the performance via multiple SNPs and human obesity-related traits as measured by body mass index (BMI), waist and hip circumferences. Our results showed that the associate statistics had type I error rates close to nominal level and were powerful for a range of effect and sample sizes. When applied to 12 candidate regions in data (*N* = 2,417) from the European Prospective Investigation of Cancer (EPIC)-Norfolk study, a region in *FTO* was found to have stronger association (rs7204609∼rs9939881 at the first intron *P* = 4.29×10^−7^) than single SNP analysis (all with P>10^−4^) and a latent quantitative phenotype was obtained using a subset sample of EPIC-Norfolk (*N* = 12,559). We believe our method is appropriate for assessment of regional association and polygenic effect on a single or multiple traits.

## Introduction

Current genetic association studies in humans, including genome-wide association studies (GWASs) [1], typically involve association of individual SNPs with a trait of interest. Notable drawbacks [2] of such an approach include multiple testing and inability to account for the correlation among SNPs in a region or treat genes as a functional unit [3]. Many attempts were made to account for correlations among SNPs, such as haplotype analysis [4], p-value or odds ratio combination [5]–[7], principal component analysis (PCA) [8], cluster [9], canonical correlation [10], data mining [11]–[14], and scan (or slide-windows) statistics [14]–[16]. Regardless the extent to which these approaches have succeeded, they are not developed for integrating multiple related traits underlying a condition or disease. For instance, type II diabetes is linked with fasting glucose, HbA1C, and glucose tolerance, among others; and obesity is another with body mass index (BMI), waist and hip circumference. Ideally, liabilities for developing diseases should be measured on quantitative dimensions [17] with available measurements [17], [18], so as to gain more statistical power and facilitate derivation of clinically relevant features [17], [19]. The case to combine multiple variants and multiple measurements is compelling and in line with the fact that an increasing number of trait-associated SNPs are identified with the challenge to implement an appropriate weighting scheme for the trait-attributing alleles.

We set to exploit association between multiple SNPs and multiple traits through a latent variable partial least squares path modeling (PLSPM) [20], [21] in a context analogous to GWAS: for the discovery sample a set of genetic variants and a latent quantitative trait are modeled through scan statistics and for the replication sample small effects of SNPs from different genes (or genomic regions) are aggregated through polygenic statistics. We examined the performance of the scan statistics with respect to type I error rate and statistical power through computer simulations. Our methods were then applied to 12 regions of GWAS data [22], [23] from the European Prospective Investigation of Cancer (EPIC)-Norfolk study.

## Methods

### Study samples

Participants in the EPIC-Norfolk study were men and women aged between 45 and 74 from Norwich and the surrounding towns and rural areas [24], [25]. In 2006, a case-cohort study was conducted in which 3,867 individuals were assayed with Affymetrix 500 K genechips among whom subcohort (*N = *2,566) was a random sample of the study cohort at baseline and cases were part of the remaining individuals with BMI≥30 kg/m^2^ (*N = *1,301). A total of 2,417 individuals in the subcohort and 1,135 cases with 446,861 SNPs passed quality control and *in silico* genotypes were obtained according to HapMap (http://www.hapmap.org) [22], [23]. An additional sample of 12,559 individuals had complete data on age, sex, BMI, waist and hip circumferences along with 12 BMI associated SNPs in or near genes *NEGR*1 (rs3101336), *SEC16B* (rs10913469), *TMEM18* (rs6548238), *ETV15* (rs7647305), *GNPDA2* (rs10938397), *BDNF* (rs925646), *MTCH2* (rs10838738), *SH2B1* (rs7498665), *FAIM2* (rs7132908), *FTO* (rs1121980), *MC4R* (rs17782313), and *KCTD15* (rs369784).

### Anthropometric measurements

The influence of body fat distribution has been linked with body shape named crudely after the fruits and vegetable(s) they resemble most [26], [27]. Studies have shown that people with a larger waist have higher risks of hypertension, type 2 diabetes and high cholesterol than those who carry excess weight on the hips [28], [29]. The combination of BMI, waist and hip circumferences is also a good predictor of cardiovascular risk and mortality [26], [29]–[32]. In this paper, nine types of body shape have been derived from the combination (Table S1) and supported by significant differences in these anthropometric traits by types and sexes. As will soon become clear, adoption of this combination as an approximate quantification of “body shape” is furnished through a latent score from formal statistical modelling. Note that the derivation differs from other possible definitions, e.g., http://en.wikipedia.org/wiki/Body_shape.

### The modeling framework

**Figure 1:**
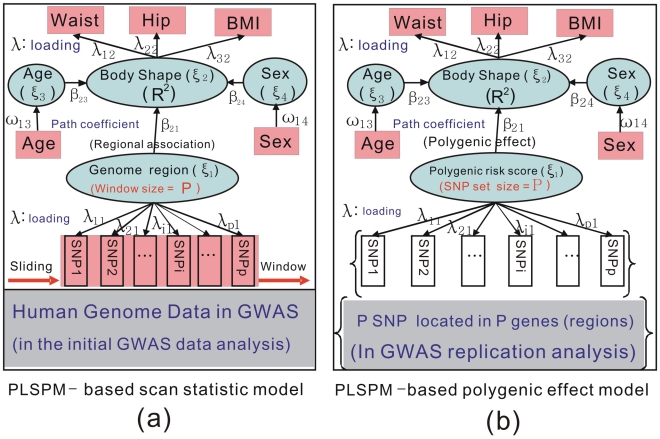
PLSPM-based models. (a) Scan statistic model, where *ξ_1_* represents genomic region containing *P* SNPs and *β_21_* the regional effect on the body shape score *ξ_1_*; (**b**) Polygenic effect model, where *ξ'_1_* represents polygenic risk score and *β'_21_* the polygenic effect. In both models, *λ*'s are the loadings while *β*'s are the path coefficients.

As hinted earlier, our framework resembles structural equation modeling (SEM) with three types of parameters defined: (1) Latent variable scores (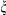) as combinations of their manifest variables obtained iteratively from an ordinary least squares (OLS)-type algorithm; (2) path coefficients (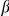
's) between dependent (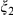) and independent latent variable (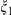) by OLS or partial least squares (PLS); (3) loadings (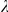
's) of each block of manifest variables with its latent variables by OLS. In this paper, the Lohmäller PLSPM algorithm was used [24], [26]. The relations between these parameters are shown in Figure 1 and used in two contexts: (1a) scan statistics are used for the detection of the genomic region (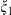) – body shape (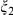) association in initial data analysis; (1b) the polygenic effect of a set of SNPs (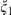) on body shape (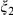) is obtained with the replication sample. More information about SEM and PLSPM is available as **Information S1**.

### Non-parametric bootstrap

As the distribution of parameters from PLS is unknown, significant test of path coefficients and loadings were furnished by bootstrap procedures [20], [33], [34]. A large, pre-specified number of bootstrap samples (5,000), each with the same number of cases as the original sample, were generated. Parameter estimation was done for each bootstrap sample, whose path coefficients or loadings can be viewed as an approximation of the sampling distribution. All bootstrap samples together provided estimators for mean and standard error of each parameter. Significance of a parameter (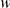) under the null hypothesis: 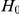
∶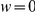 and the alternative 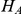
∶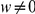 was tested via a normal test in the form 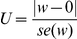 (e.g., 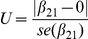) where 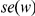 is the bootstrapped standard error [20], [21].

### Interpretation

Let 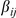 = the path coefficient between the *i*-th and the *j*-th latent variable and 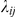 = loading between the *i*-th manifest variable and the *j*-th latent variable. The interpretation can then be facilitated according to Figure 1: (1) path coefficient (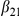) in the structure (inner) model represents an overall effect of the genome region or polygenic effect of a SNPs set (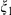) on body shape (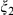); (2) 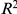 is the proportion of variance explained; (3) With path coefficients and loading obtained from the standardized variables, their product in a given path is a measure of the effect of a specific SNP on a single trait or body shape (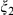). For example, the effect of SNP2 on body shape (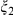) is 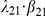, and that on BMI is 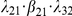; (4) Body shape score (BSS), as a combination of waist, hip and BMI with weights 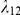, 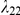, and 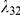, represents a latent quantitative phenotype of body shape such that 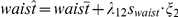, 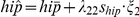, 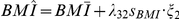 with the body type determined by (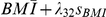) and (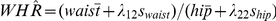 according to their thresholds (Table S1), and (5) the latent polygenic liability (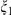) aggregated by small effects of DNA variants in different genome regions with their weights 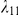, 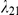, …, 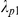 is the polygenic risk score (PRS) of the SNP set (Figure 1b).

### Simulation

Simulations were conducted as follows: (1) HapMap phase II *CEU* data at the brain-derived neurotrophic factor (*BDNF*) region (Chr 11∶27633610..27692970 with 24 SNPs) were used to generate the simulated genotypic data; (2) Based on (1), a large sample of 500,000 individuals was obtained via software **gs** 2.0 [35] with the 6^th^ SNP being the causal variant; (3) Quantitative genetic data was generated according to a trivariate normal distribution 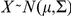, where 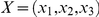 is the random vector (waist, hip, BMI) for “apple-shaped” types (N = 355) in EPIC-Norfolk GWAS subcohort with their sample mean 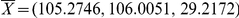 and covariance 
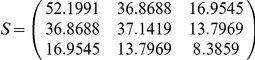
_._ Assume that the causal SNP had no effect on body shape (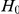), 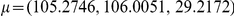 for all three genotypes (GG, GA, and AA) and that the causal SNP effects on waist not on hip, and the single allele effect size on BMI is *δ* kg/m^2^ (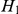), 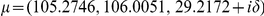, where *i = 0,1,2* for GG, GA and AA, respectively. The range of 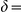 (0.10, 0.15, 0.20, 0.25, 0.30) was estimated by published data on genetic predisposition score [18]. Given the increment 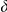 on BMI, estimation of waist under fixed hip was obtained by 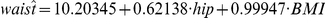 (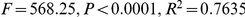) established by the same “apple-shaped” data in the EPIC-Norfolk GWAS; (4) Genotypic data were simulated under various sample sizes from the simulated *CEU* population (500,000 individuals), and quantitative genetics models with the given ***δ*** were created by the R **mvtnorm** package. The window size had 10 SNPs from the 3^th^ to the 12^th^ SNP. Under *H*_0_, 10,000 simulations given various sample sizes were conducted to assess the type I error. Under *H*_1_, for each model and a given δ, 10,000 simulations were conducted under various sample sizes to assess power. The procedures were implemented with Linux and the R **plspm** package. Both **mvtnorm** and **plspm** packages are available from CRAN. (http://cran.r-project.org/)

### Analysis of the EPIC-Norfolk data

Scan statistics were built through the subcohort for association between the 12 regions and body shape, and to contrast with a SNP-wise single trait test performed by linear regressions (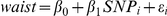, 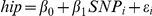, 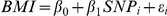) according to sizes of sliding windows of 1 to 15 SNPs, and the *α*-level was defined as 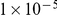 according to the literature [4] for region-based analysis. Polygenic effects on single or latent traits with the PLSPM polygenic statistics were obtained and compared with unweighted sum of BMI-increasing alleles [18] and we also assessed whether 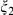 is an appropriate latent quantitative measurement.

## Results

### Simulation

**Figure 2:**
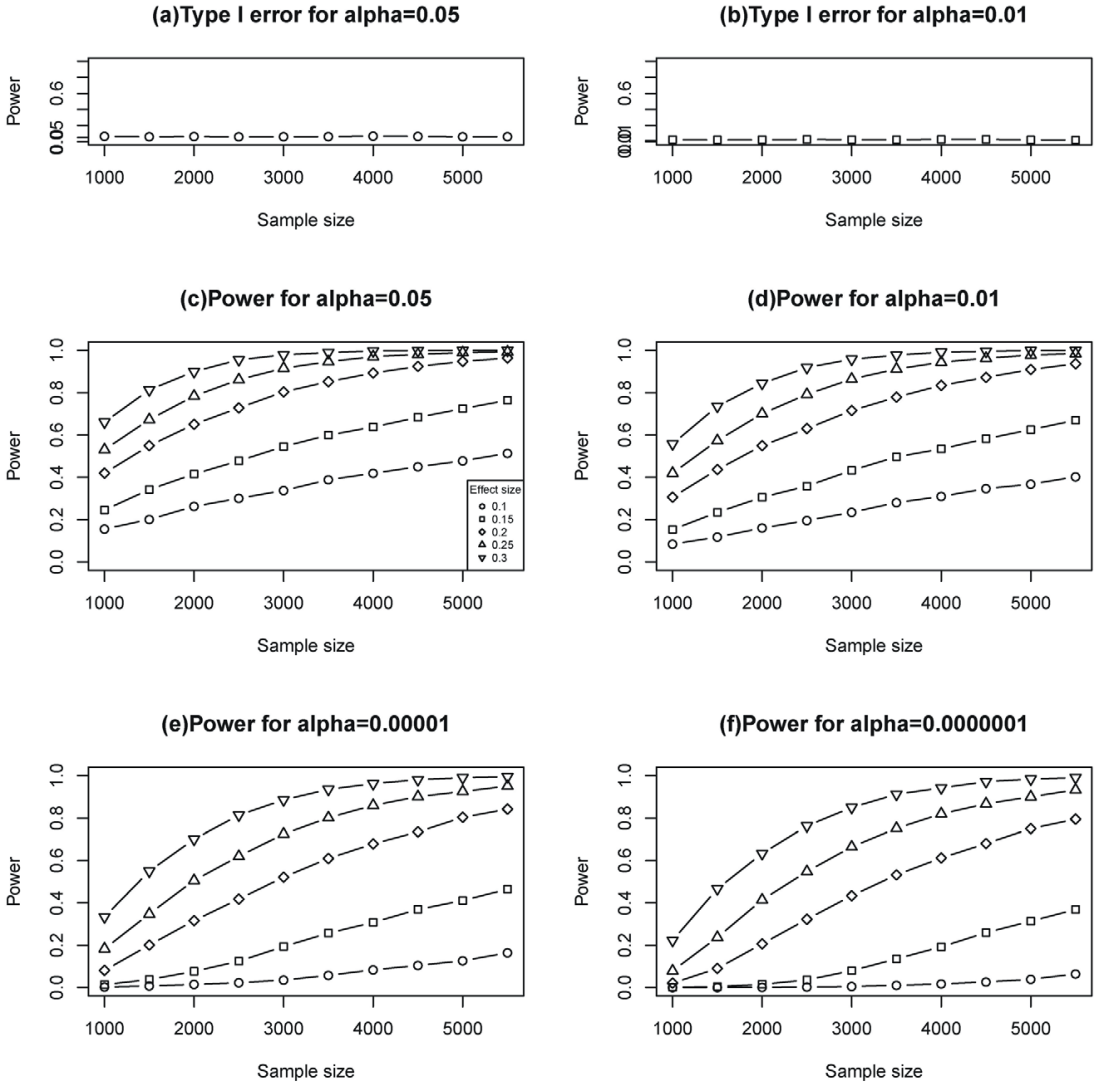
Simulation results of type I error and power for scan statistic model.

As shown in Figure 2, the type I error rates of the scan statistics were close to nominal levels (0.01, 0.05) as a function of sample sizes (2a, 2b). Power monotonically increases with sample size, effect size (*δ*), or nominal level (*α*) (2c–2f). Even with a very small *α*, for effect size greater than 0.15 and the sample size of up to 4,500, the scan statistics remained to have >80% power (2e, 2f).

### Analysis of the EPIC Norfolk data

#### Single trait results

The model provided the usual association results for single trait adjusted for sex and age including effect size estimate, proportion of variance explained and statistical significance. Results on BMI, waist and hip circumferences were also similar for PRS. Shown in Figure S1 and Table S2 are SEM and results of the 12 SNPs in the 12 gene regions adjusted for sex and age for single trait (a1,b1,c1) as with distribution of their PRS and cumulative effects of these variants (a2,b2 c2). More details can be found in **Information S2**.

#### Multi-trait results

**Figure 3:**
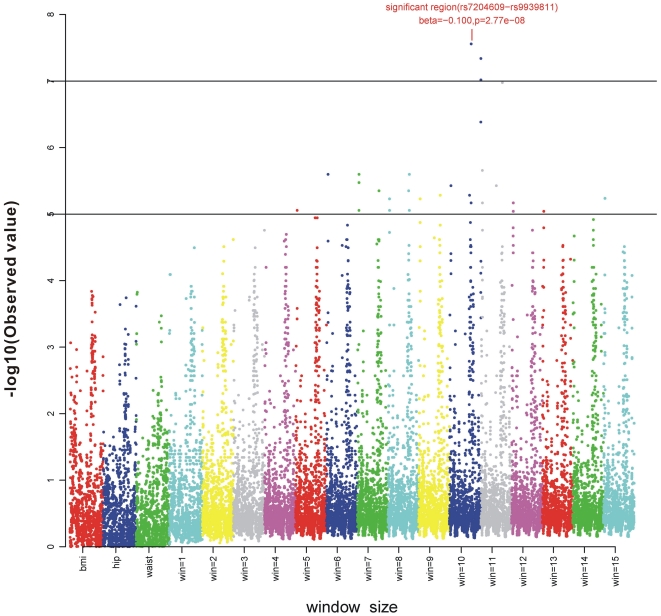
Manhattan plot for single and multiple traits in the 12 gene regions.

**Figure 4:**
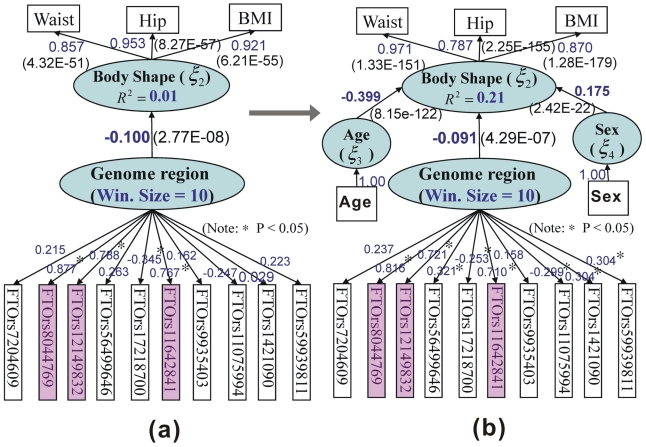
Fitted models for the specific region within the first intron 1 of *FTO* gene without (a) and with (b) adjustment for covariates.

As shown in Figure 3, none of the SNPs were significant at 10^−4^ level according to single-SNP –single-trait tests nor according to sliding window sizes of 1–4 SNPs at the 10^−5^ level, but smaller p values were obtained for window sizes of 5–11 and 12–15 SNPs. Of particular interest was rs7204609∼rs9939881 at the first intron of *FTO* with 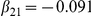, *P* = 4.29×10^−7^ for a sliding window of size 10; its model structure is shown in Figure 4. The standardized overall effect (95% CI) of the genome region on body shape was −0.100 (−014- −0.08) without adjustment for sex and age, and −0.09 (−0.13- −0.07) with adjustment. The effect (95%CI) of a specific SNP on body shape or on a single trait are available −0.09 (−0.08- −0.08) and 0.07 (−0.06- −0.05) after adjusting for sex and age, respectively for rs58044769. These results suggest that the location of the causal variant in the 10-SNP loading vector is likely between the rs58044769 and rs11642841 (the sixth SNP).

**Figure 5:**
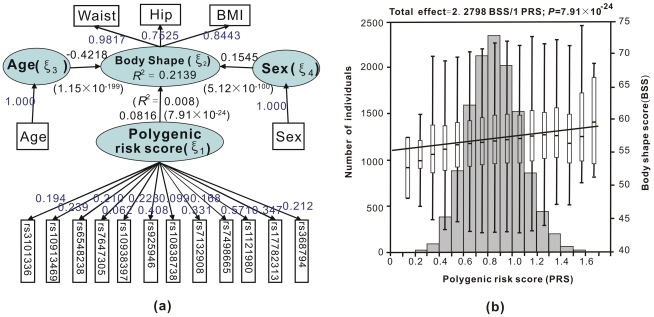
Fitted model for the 12 SNPs from the 12 gene regions with adjustment for sex and age for multiple traits (a) as with distribution of its PRS and cumulative effects of these variants (b).

Figure 5a and Table 1 show models and results of the 12 SNPs in the 12 gene regions adjusted for sex and age, where the standardized effect (λ_SNP_⋅*β_21_*) (95%CI) per allele on body shape was 0.08 (0.07–0.10, P = 7.91×10^−24^). The proportion of variance explained was 0.8% by PRS. All genetic variants showed associations with body shape, though some loadings of the SNPs were not significant at *α* = 0.05 (Table 1). There were substantial variations in standardized effects of each SNP with the largest being rs1121980 (*FTO*) and rs925646 (*BDNF*) for all the four traits, followed by rs6538238 (*TMEM18*), rs17782313 (*MC4R*) for BMI and hip; rs17782313 (*MC4R*), rs7132908 (*FAIM2*) for waist and body shape. Non-standardized effect sizes were largest with rs1121980 (*FTO*) (0.39), but smallest with rs7647305 (*ETV5*) (0.05) (see also Figures 5 and S1).

**Table 1:** Loadings, p values, indirect and overall effects of 12 SNPs, PRS on body shape with adjustment for sex and age.

SNP/PRS or measurements	Gene	Body shape (*β*_21_ = 0.0816, *P* = 7.91×10^−24^)			
		Loading (λ)	*P* value	Indirect effect (*λ⋅β*_21_)	Overall effect
rs3101336	*NEGR1*	0.1939	0.0635	0.0158	0.1362
rs10913469	*SEC16B*	0.2386	0.0198	0.0195	0.2033
rs6548238	*TMEM18*	0.2097	0.0406	0.0171	0.1892
rs7647305	*ETV5*	0.0622	0.5452	0.0051	0.0515
rs10938397	*GNPDA2*	0.2217	0.0309	0.0181	0.1513
rs925946	*BDNF*	0.4080	1.07E-05	0.0333	0.3004
rs10838738	*MTCH2*	0.0987	0.3221	0.0081	0.0699
rs7132908	*FAIM2*	0.3305	0.0017	0.0270	0.2302
rs7498665	*SH2B1*	0.1684	0.1084	0.0137	0.1168
rs1121980	*FTO*	0.5714	1.08E-10	0.0466	0.3908
rs17782313	*MC4R*	0.3466	0.0005	0.0283	0.2788
rs368794	*KCTD15*	0.2117	0.0352	0.0173	0.1538
PRS			7.91E-24	0.0816	2.2798
waist		0.9817	0.00E+00		
Hip		0.7525	0.00E+00		
BMI		0.8443	0.00E+00		

Shown in Figure 5b is the distribution of PRS and cumulative effects of these variants, from which we made the following observations: (1) PRS was normally distributed, with ranges of 0.05–1.69 for body shape, with the majority (68.27%) of individuals (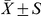) also showing similar patterns of PRS (0.86±0.21); (2) for each level of PRS the distribution of body shape had similar pattern according to boxplots, generally normally distributed with range 0.4–1.3 for PRS but skewed with <0.4 or >1.3; (3) The means of body shape score increased linearly with PRS, with on average each additional unit associated with increments (*P*) of 2.28 (7.91×10^−24^).

**Figure 6:**
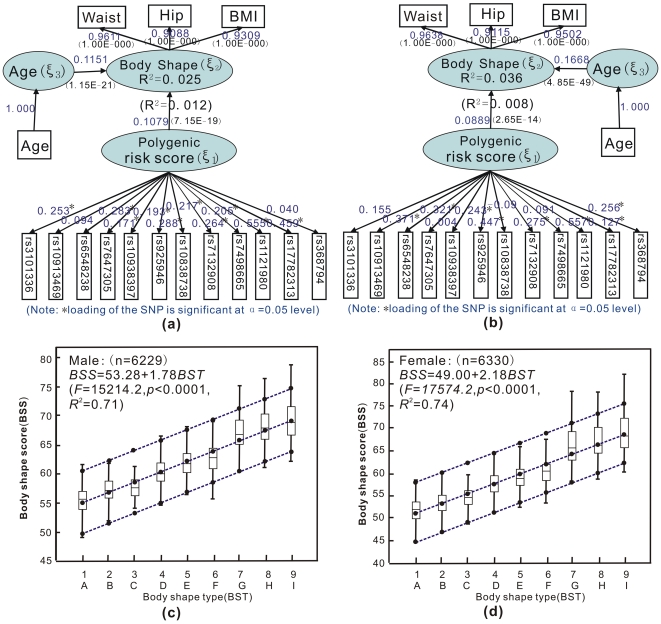
SEM of body shape score in the EPIC-Norfolk replication samples (a,b), the linear regression between BSS and body shape types (c,d).

Shown in Table 2 and Figure 6 are the distribution of body shape types and characteristics of body shape score in the EPIC-Norfolk replication samples, from which several observations can be made. (1) types (men%, women%) were predominantly 1 (29.30%, 33.87%), 4 (31.58%, 19.35%) and 5 (16.55%, 11.61%). There was significant sex difference of overall body shape types (*χ* ^2^ = 1556.8, *P*<.0001), especially in types 4, 5, 6, 9, 2, 3; (2) for both men and women, along with the risk of obesity, body shape score was seen to be monotonically increasing from types 1 to 9 (Table 2 and Figures 6c, 6d), with significant differences between given two types (*F* = 1994.80, *P*<0.0001 for men, *F* = 2468.78, *P*<0.0001 for women, both with p<0.05 according to *SNK* test). Linear regression between the scores and types had good fit for men (*F* = 15214.2, *P*<0.0001, *R* ^2^ = 0.71) and women (*F* = 17574.2, *P*<0.0001, *R* ^2^ = 0.74) (Figures 6c, 6d), suggesting body shape score is an excellent measure; (3) BSS follows an approximate normal distribution (see Figure S2); (4) The estimated BMIs and waist-hip ratios (WHRs) were 29.52 and 0.97 for men, 30.25 and 0.83 for women, respectively. Polygenic effects of the 12 SNPs contributed to type 5 on the basis of the nine (Table S1) in both men and women.

**Table 2:** Distribution of body shape types and characteristics of body shape score (BSS) by sex in the EPIC-Norfolk study.

Body shape types	Symbol	Men (*F* = 1916.50, *P*<0.0001)				Women (*F* = 2457.32, *P*<0.0001)			
		*n*	*%*	*Mean* ± *SD*	95% *CI*	*n*	*%*	*Mean* ± SD	95% *CI*
Chilli	**A (1)**	1825	29.30	55.25±2.57	(55.13,55.37)	2144	33.87	51.57±2.69	(51.47,51.68)
Chilli pear-apple	**B (2)**	196	3.15	57.13±2.11	(56.83,57.43)	455	7.19	53.17±2.58	(52.93,53.41)
Chilli apple	**C (3)**	46	0.74	57.45±2.21	(56.82,58.08)	209	3.30	54.33±2.51	(53.99,54.67)
Pear	**D (4)**	1967	31.58	60.31±2.31	(60.21,60.41)	1225	19.35	57.69±2.56	(57.55,57.83)
Pear-apple	**E (5)**	1037	16.65	61.87±2.31	(61.73,62.01)	735	11.61	58.88±2.51	(58.70,59.06)
Apple	**F (6)**	348	5.59	62.60±2.66	(62.32,62.88)	552	8.72	60.16±2.67	(59.94,60.38)
Big pear	**G (7)**	210	3.37	67.41±3.94	(66.87,67.94)	288	4.55	66.46±5.37	(65.84,67.08)
Big pear-apple	**H (8)**	334	5.36	68.43±3.65	(68.04,68.82)	325	5.13	67.67±5.13	(67.11,68.23)
Big apple	**I (9)**	266	4.27	69.50±4.19	(69.00,70.00)	397	6.27	69.31±5.50	(68.77,69.85)
Total		6229	100.00	60.16±4.94	(60.04,60.28)	6330	100.00	57.17±6.51	(57.01,57.33)

## Discussion

A latent variable PLSPM framework is outlined for association of multiple SNPs with multiple traits, the behavior of such an association was investigated by simulation study through type I error rate and power. Meanwhile, a polygenic statistic was developed for quantification of a polygenic effect by appropriately weighting trait-attributing alleles. These methods were applied to the study of obesity-related variables in the EPIC-Norfolk study for which a latent score was obtained. Below we compare these with available methods, discuss implications of our findings as with other issues involved and indicate some further work.

Compared to SEM, PLSPM is robust to multicollinearity commonly encountered in GWAS data (such as strong linkage disequilibrium between SNPs and high correlation between traits). It is a “soft modelling” approach requiring very few distributional assumptions, variables can be numerical, ordinal or nominal, and no need for normality assumptions, while covariance-based SEM is a “hard modeling” with heavy distributional assumptions [20], [21]. Through simulation, the scan statistics gave a good approximation of the type I error rate and proved powerful for novel region-based latent quantitative traits analysis, even with very high significant level and a modest single SNP effect size. Our result also agreed with the literature regarding the optimality of a 10-SNP window [4]. Our scan statistics are embedded with the “thinking quantitatively framework” [17] such that there is a theoretical quantitative trait for each qualitative trait and normally distributed polygenic liabilities. Their advantages are as follows: First, it can capture the association between a genomic region and a latent quantitative phenotype of disorder (or trait) all in continuous quantitative dimensions. Second, the model structure provides abundant information for interpretation. Third, fine region of the causal SNP can be located by the loading vector of SNPs in the window (the potential causal variant is probably located between rs58044769 and rs11642841). The latent score of obesity-related variables is a synthetic quantitative phenotype which effectively combines waist, hip and BMI to reflect the risk of obesity in accordance with increasing WHR given increasing BMI. Its derivation is a motivating example for many other disorders and traits, such as diabetes, heart disease and metabolic syndrome.

Analysis of the EPIC-Norfolk discovery sample involving 12 gene regions suggested that the scan statistics are more powerful than single SNP – single trait tests with the size 10 providing the strongest evidence. In particular, the region (rs7204609∼rs9939811) within the first intron 1 of *FTO* gene is of interest, as with some of the reported obesity-susceptibility SNPs near or in the 12 genes [18]. We would like to highlight the utility of PRS. It refers to a set of DNA variants in different genome regions associated with a trait, termed previously as polygenic susceptibility score [36], genomic profiles [37], SNP set [38], aggregate risk scores [39] or genetic predisposition score [18]. Their apparent drawback is the lack of an appropriate scheme for weighting. PRS not only weights the individual risk alleles by the loading vector of the SNP set but also furnishes association analysis between PRS and a latent quantitative phenotype (BSS). Our data showed that PRS was normally distributed, which is consistent with the notion that a theoretical quantitative trait correspond to normally distributed polygenic liabilities (see Figure 5b, Figure S1-(a2,b2,c2)) [17]. Unlike the unweighted estimator, it is also coherent and accurate. For instance, total effects of PRS or a specific SNP on the single trait (BMI, waist or hip circumferences) and on the latent quantitative phenotype (body shape) can be compared by the standardized path coefficient or the product of loading and path coefficients along the path, respectively. The non-standardized path coefficient or the product of loading and path coefficients, total effects of a specific SNP on a single trait and on the latent quantitative phenotype can also be obtained. The mean BMI, waist circumference, hip circumference and body shape score increased in a linear fashion as the PRS increases. The effect of PRS on body shape type can be derived.

A reviewer has indicated previous work on multiple linked quantitative trait loci (QTLs) [40], [41] that bear some spirit to our use of multiple SNPs. Together with the academic editor they have expressed concerns over the possible impact of population stratification. Fortunately, with availability of genomic data such a concern can be relieved with multiple markers directly [42] or via summary statistics from principal components analysis [43]. The EPIC-Norfolk GWAS has contributed to a variety of consortia, for which the inflation factor derived from per SNP association statistics is always close to one. This is likely to be the result of both homogenous sample and exclusion of outliers at the quality control stage. We believe the analysis as conducted in this report will not be affected. However, in general, it may be necessary to include summary statistics such as principal components as covariates in the model.

A reviewer has questioned the adequacy of body shape as with PLS with a view that body share should be supported by various other measurements such as limb lengths, shoulder widths, etc. However, our interest lies more in utilizing the anthropometric traits from a population study for investigation of health risks. Indeed our results showed that BSS is approximately normal (Figure S2) and serves as an excellent measurement of body shape types (Figures 6c, 6d). The use of latent trait is also consistent with Fisher's derivation of polygenic effect [17]. At the time the paper was submitted for publication, a form of PLS has appeared for multiple markers [44].

There will be several lines of further research. Firstly, there is an important need to examine the precise nature of regional or polygenic effect on a single trait or a collection of traits, as it may involve both polygenic and pleiotropic effects. This is also the case with GWAS. Long before this work when we reported work using SEM to differentiate pleiotropic effect on obesity-related traits in a GIANT consortium (http://www.broadinstitute.org/collaboration/giant/index.php/Main_Page) teleconference, a colleague instantly questioned the feasibility across the whole consortium. Secondly, the scan statistics seemed slightly anticonservative and a parametric counterpart is preferable. Thirdly, it will be desirable to catch both linear and nonlinear effects between genome region and latent quantitative trait.

## Supporting Information

Figure S1 SEM of the 12 SNPs in the 12 gene regions adjusted for sex and age for single trait (a1,b1,c1) as with distribution of their PRS and cumulative effects of these variants (a2,b2 c2).
(TIF)

Figure S2 The distribution of body shape score (BSS).
(TIF)

Table S1 Nine types of human body shape defined by BMI combination with WHR.
(DOC)

Table S2 Loadings, P values, indirect and overall effects of 12 SNPs and PRS on BMI, waist and hip with adjustment for sex and age.
(DOC)

Information S1 Some theoretical results.
(DOC)

Information S2 Single trait results from the EPIC-Norfolk replication sample.
(DOC)
