# Supplementary figures and images for: A Latent Variable Partial Least Squares Path Modeling Approach to Regional Association and Polygenic Effect with Applications to a Human Obesity Study
Source: PLoS One. 2012 Feb 27;7(2):e31927. doi: 10.1371/journal.pone.0031927 (PMC3288051; doi:10.1371/journal.pone.0031927)

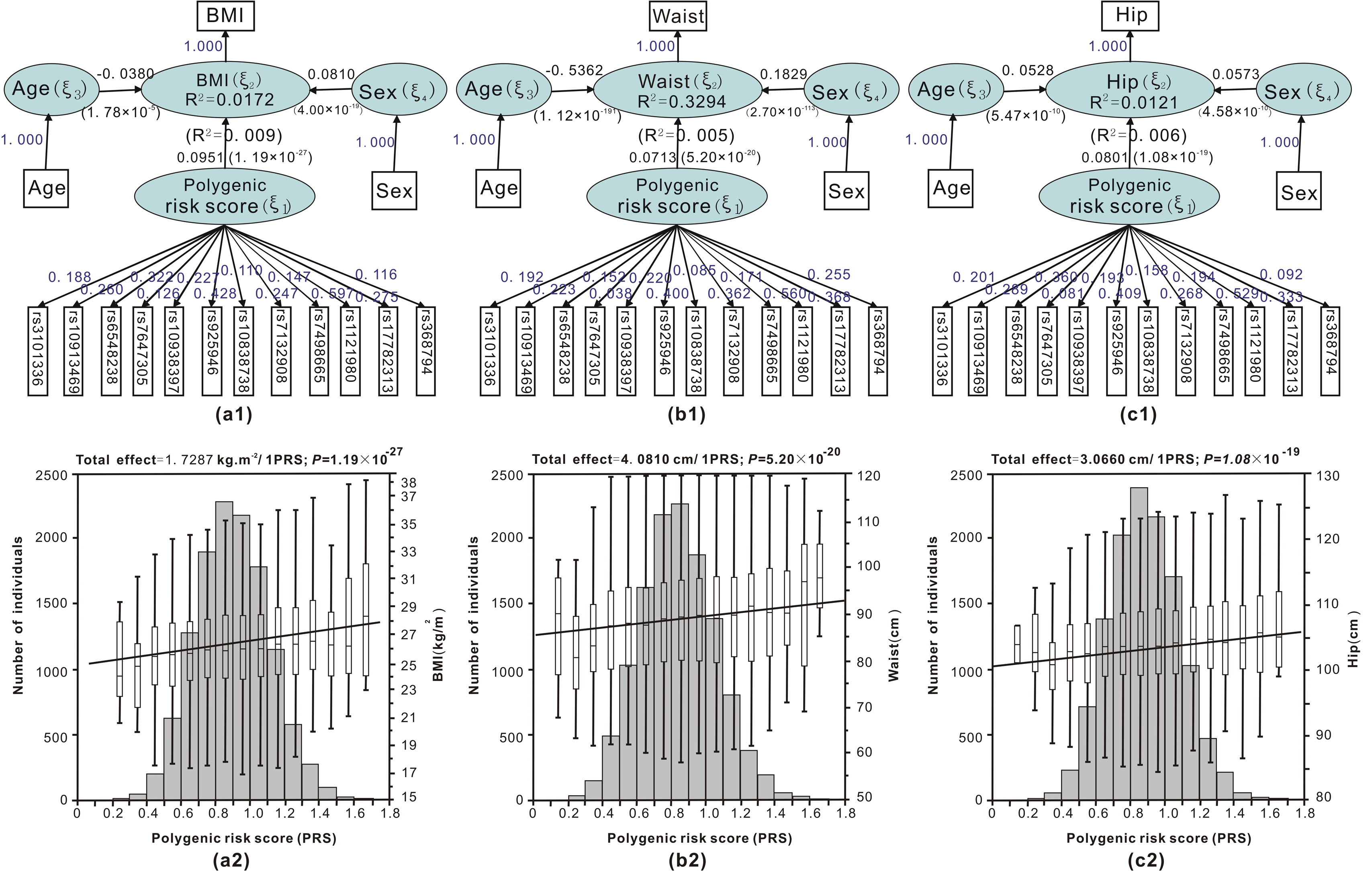

Supplement: Figure S1 — SEM of the 12 SNPs in the 12 gene regions adjusted for sex and age for single trait (a1,b1,c1) as with distribution of their PRS and cumulative effects of these variants (a2,b2 c2). (TIF) [file pone.0031927.s001.tif]

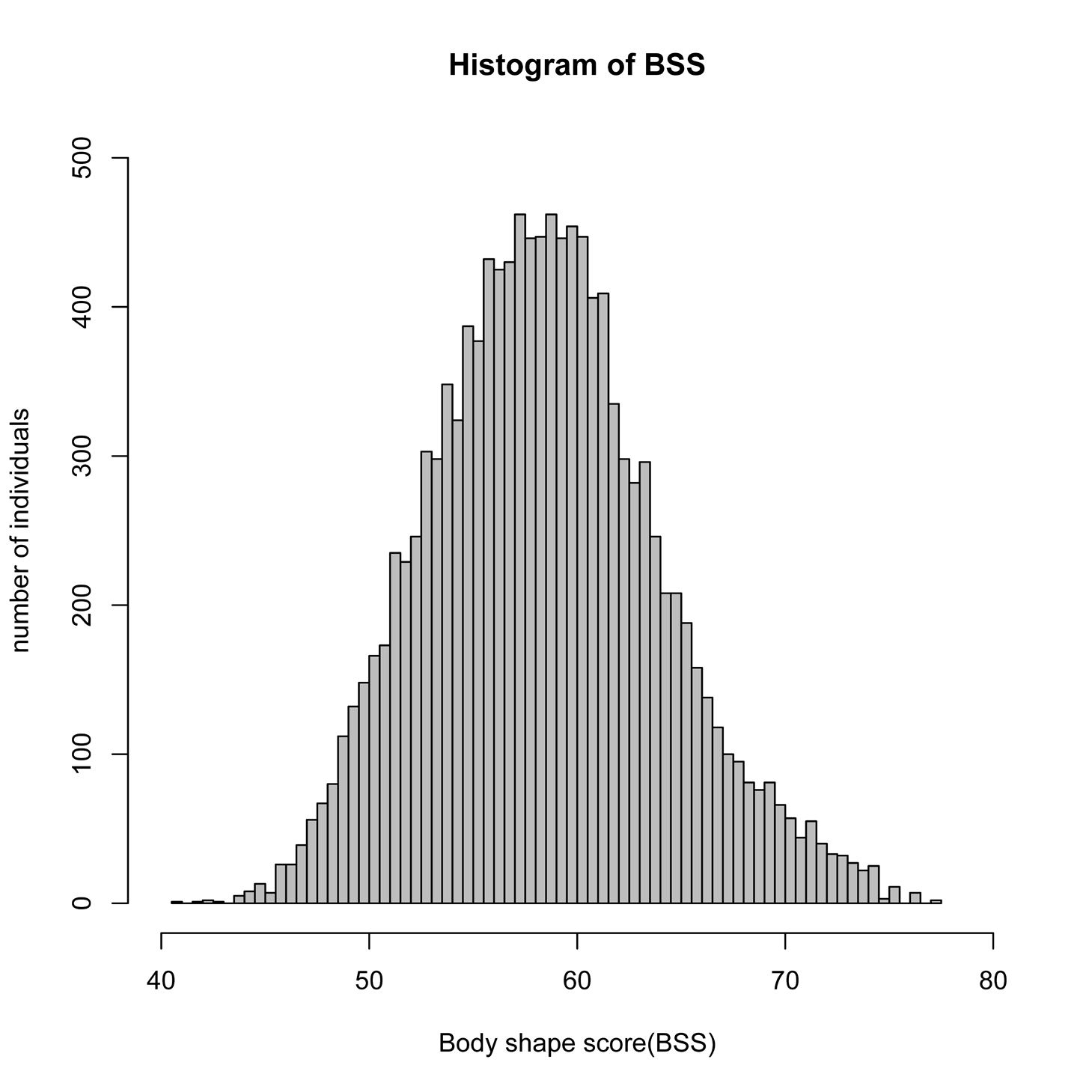

Supplement: Figure S2 — The distribution of body shape score (BSS). (TIF) [file pone.0031927.s002.tif]
