# Supplementary material for: A Latent Variable Partial Least Squares Path Modeling Approach to Regional Association and Polygenic Effect with Applications to a Human Obesity Study
Source: PLoS One. 2012 Feb 27;7(2):e31927. doi: 10.1371/journal.pone.0031927 (PMC3288051; doi:10.1371/journal.pone.0031927)
Supplement: Table S1 — Nine types of human body shape defined by BMI combination with WHR. (DOC) [file pone.0031927.s003.doc]

Table S1. Nine types of human body shape defined by BMI combination with WHR

| BMI（*kg/m*^2^）  WHR(*cm*) | | 0~ | 25~ | 30~ |
| --- | --- | --- | --- | --- |
| male | 0.00~ | Chilli (1) | Pear (4) | Big pear (7) |
|  | 0.95~ | Chilli pear-apple (2) | Pear-apple (5) | Big pear-apple (8) |
|  | 1.00~ | Chlii apple (3) | Apple (6) | Big apple (9) |
| female | 0.00~ | Chilli (1) | Pear (4) | Big pear (7) |
|  | 0.80~ | Chilli pear-apple (2) | Pear-apple (5) | Big pear-apple (8) |
|  | 0.85~ | Chlii apple (3) | Apple (6) | Big apple (9) |
