# Supplementary material for: A Latent Variable Partial Least Squares Path Modeling Approach to Regional Association and Polygenic Effect with Applications to a Human Obesity Study
Source: PLoS One. 2012 Feb 27;7(2):e31927. doi: 10.1371/journal.pone.0031927 (PMC3288051; doi:10.1371/journal.pone.0031927)
Supplement: Table S2 — Loadings, P values, indirect and overall effects of 12 SNPs and PRS on BMI, waist and hip with adjustment for sex and age. (DOC) [file pone.0031927.s004.doc]

Table S2. Loadings, P values, indirect and overall effects of 12 SNPs and PRS on BMI, waist and hip with adjustment for sex and age

| SNP/PRS | Gene | BMI (*β*_21_=0.0951, *P*=1.19×10^-27^) | | | | Waist (*β*_21_=0.0713, *P*=5.20×10^-20^) | | | | Hip (*β*_21_=0.0801, *P*=1.08×10^-19^) | | | |
| --- | --- | --- | --- | --- | --- | --- | --- | --- | --- | --- | --- | --- | --- |
|  |  | *λ* | *P* | *λ⋅β*_21_ | Overall Effect | *λ* | *P* | *λ⋅β*_21_ | Overall effect | *λ* | *P* | *λ⋅β*_21_ | Overall effect |
| rs3101336 | *NEGR1* | 0.1883 | 0.0369 | 0.0179 | 0.0997 | 0.1924 | 0.0882 | 0.0137 | 0.2466 | 0.2010 | 0.0459 | 0.0161 | 0.1843 |
| rs10913469 | *SEC16B* | 0.2600 | 0.0033 | 0.0247 | 0.1671 | 0.2225 | 0.0484 | 0.0159 | 0.3460 | 0.2887 | 0.0083 | 0.0231 | 0.3212 |
| rs6548238 | *TMEM18* | 0.3225 | 6.03E-5 | 0.0307 | 0.2195 | 0.1518 | 0.1756 | 0.0108 | 0.2500 | 0.3594 | 0.0002 | 0.0288 | 0.4235 |
| rs7647305 | *ETV5* | 0.1256 | 0.1688 | 0.0119 | 0.0784 | 0.0375 | 0.7446 | 0.0027 | 0.0566 | 0.0811 | 0.4466 | 0.0065 | 0.0876 |
| rs10938397 | *GNPDA2* | 0.2276 | 0.0128 | 0.0216 | 0.1172 | 0.2201 | 0.0051 | 0.0157 | 0.2742 | 0.1929 | 0.0665 | 0.0155 | 0.1719 |
| rs925946 | *BDNF* | 0.4275 | 6.38E-7 | 0.0407 | 0.2374 | 0.3956 | 0.0003 | 0.0282 | 0.5315 | 0.4085 | 2.07E-5 | 0.0327 | 0.3927 |
| rs10838738 | *MTCH2* | 0.1164 | 0.2146 | 0.0111 | 0.0622 | 0.0848 | 0.4669 | 0.0060 | 0.1096 | 0.1577 | 0.1336 | 0.0126 | 0.1459 |
| rs7132908 | *FAIM2* | 0.2466 | 0.0072 | 0.0235 | 0.1295 | 0.3617 | 0.0012 | 0.0258 | 0.4597 | 0.2683 | 0.0086 | 0.0215 | 0.2440 |
| rs7498665 | *SH2B1* | 0.1471 | 0.1230 | 0.0140 | 0.0770 | 0.1706 | 0.1179 | 0.0122 | 0.2160 | 0.1937 | 0.0673 | 0.0155 | 0.1755 |
| rs1121980 | *FTO* | 0.5966 | 6.54E-17 | 0.0567 | 0.3078 | 0.5595 | 1.68E-9 | 0.0399 | 0.6983 | 0.5289 | 5.50E-9 | 0.0424 | 0.4724 |
| rs17782313 | *MC4R* | 0.2753 | 0.0018 | 0.0262 | 0.1670 | 0.3679 | 0.0005 | 0.0262 | 0.5400 | 0.3329 | 0.0010 | 0.0267 | 0.3496 |
| rs368794 | *KCTD15* | 0.1661 | 0.2246 | 0.0158 | 0.0910 | 0.2548 | 0.0252 | 0.0182 | 0.3378 | 0.0920 | 0.3777 | 0.0074 | 0.0873 |
| PRS |  |  | 1.19E-27 | 0.0951 | 1.7287 |  | 5.20E-20 | 0.0713 | 4.0810 |  | 1.08E-19 | 0.0801 | 3.0660 |
