# Supplementary material for: A Latent Variable Partial Least Squares Path Modeling Approach to Regional Association and Polygenic Effect with Applications to a Human Obesity Study
Source: PLoS One. 2012 Feb 27;7(2):e31927. doi: 10.1371/journal.pone.0031927 (PMC3288051; doi:10.1371/journal.pone.0031927)
Supplement: Information S1 — Some theoretical results. (DOC) [file pone.0031927.s005.doc]

**Information S1. Some theoretical results**

Structural equation modeling (SEM) is a comprehensive statistical approach to testing hypotheses about relations among observed and latent variables which are unobserved and implied by the covariances among two or more indicators [1]. It involves:

1. Model specification. Relations between variables can be of three types: 1). association, which is nondirectional and identical to correlation between variables; 2), direct effect, which is a directional relation between two variables; 3). indirect effect, which is the effect of a dependent variable through one or more intervening or mediating variables. Typically, a measurement model associate the observed with latent variables and a structural model delineates the relations among latent variables and observed variables that are not indicators of latent variables.
2. Model identification. Parameters in the model are usually specified via covariance and treated as from multivariate normal distribution, so that parameters can be estimated in a framework like maximum likelihood.
3. Evaluation and modification. The adequacy of the model is assessed by goodness of fit statistics between possible alternative models.
4. Interpretation. The effect size or statistical significance of parameters with respect to direct or indirect effect can be facilitated by asymptotic theory or computer-intensive methods such as bootstrap. A set of path tracing rules can be used similar to path analysis: 1) one can trace backward up an arrow (🡐) and then forward along the next (🡒), or forwards from one variable to the other (🡒), but never forward and then back; 2) one can pass through each variable only once in a given chain of paths; 3) no more than one bidirectional arrows (🡘) can be included in each path-chain.

In contrast, partial least squares path modeling (PLSPM) uses a PLS approach to SEM[2]:

1. It models relations via structural model (inner) and measurement model (outer)[3,4]. The formulation of measurement model vary with the direction of the relationships between the latent variables and the corresponding manifest variables by accommodating both reflective (effects) and formative (causes) indicators[2,3]. In a reflective model the block of manifest variables related to a latent variable is assumed to measure a unique underlying concept. Because of this, it should be homogeneous and unidimensional, e.g., the first eigenvalue of its correlation matrix is higher than 1. In a formative model, each manifest variable or sub-block of manifest variables represents a different dimension of the underlying concept.
2. It is variance or component-based rather than covariance-based. As a soft modeling” approach, it requires few distributional assumptions and allows for numerical, ordinal or nominal variables. It employs an iterative algorithm to separately solve out the blocks of the measurement model and then estimates the path coefficients in the structural model. Consequently, it explains at best the residual variance of the latent variables and potentially of the manifest variables in any regression run in the model. It is exploratory and does not aim to reproduce the sample covariance matrix. Latent variable and manifest variables are standardized in a reflective model and is hardly affected by multicollinearity. It employs a combination of manifest variables with a multidimensional form to minimize residuals in structural relationships for a higher value of.
3. It is possible to assess model fit and the statistical significance of parameters via cross-validation methods like bootstrap.
4. As will be shown below, the interpretation resembles SEM.

Illustrated in Figure 1 is the collective effect of SNPs on obesity-related traits including BMI, waist and hip circumferences. The associate latent variables represent genomic region, body shape, age and sex, respectively, with being the parameters or loadings linking the latent variables with indicators andthe effects of latent variables. The inner model consists of 🡪 and outer model links these latent variables with manifest variables (SNPs and waist, hip, BMI). A manifest variable in a reflective model has corresponding measurement model to be a linear function of its latent variable (*ξ*) and residual () such that

The direct effectsand have their own interpretations as are used to form indirect effects such asand for SNP_1_ to latent variables and waist, respectively, with the chain of paths SNP_1_🡐🡒🡒waist as a legitimate path. As in Figure 1, body shape () are associated with Dynamic versions are available through scan statistics ofSNPs in the genome, as does PRS () aggregate the small effects of SNPs from different genes (genomic regions).

References

1. Hoyle RH (1995) Structural Equation Modeling Approach: Basic Concepts and Fundamental Issues. In: Hoyle RH, editors. Structural Equation Modeling: Concepts, Issues, and Applicatio. SAGE. pp. 1-15.

2. Esposito VV, Chin WW, Henseler J,Wang H (2010) Handbook of Partial Least Squares: Concepts, Methdos and Applications. Berlin Heidelberg: Springer.

3. Henseler J, Ringle CM, Sinkowics RR (2009) The Use of Partial Least Squares Path Modeling in International Marketing. Advin Intern Marketing 20: 277-319.

4. Fornell C, Bookstein FL (1982) 2 Structural Equation Models - Lisrel and Pls Applied to Consumer Exit-Voice Theory. Journal of Marketing Research 19: 440-452.
