# Supplementary material for: A Latent Variable Partial Least Squares Path Modeling Approach to Regional Association and Polygenic Effect with Applications to a Human Obesity Study
Source: PLoS One. 2012 Feb 27;7(2):e31927. doi: 10.1371/journal.pone.0031927 (PMC3288051; doi:10.1371/journal.pone.0031927)
Supplement: Information S2 — Single trait results from the EPIC-Norfolk replication sample. (DOC) [file pone.0031927.s006.doc]

**Information S2. Single trait results from the EPIC-Norfolk replication sample**

Figure S1 (a1, b1, c1) and Table S2 show models and results of 12 SNPs on a single trait adjusted for sex and age, so that: 1) standardized overall effects (95%CI) of per allele on BMI, waist, hip, and body shape were 0.10 (0.08-0.11), 0.07 (0.06-0.09), 0.08 (0.07-0.10) with P values of 1.19×10^-27^, 5.20×10^-20^, 1.08×10^-19^, respectively. The non-standardized overall effects were 1.73*kg⋅m*^-2^, 4.08*cm*, 3.07*cm*, respectively. 2) proportions of variance explained were 1.72%, 32.94%, 1.21% by PRS with sex and age respectively compared to 0.9%, 0.5%, 0.6% without sex and age. 3) all genetic variants showed associations with the three traits, though some loadings of the SNPs were not significant at *α*=0.05 (Table S1). There were substantial variations on standardized effects of a SNP (λ_i1_*β_21_*, *i=1,…,P*) with the largest being rs1121980 (*FTO*) and rs925646 (*BDNF*) for all the four traits, followed by rs6538238 (*TMEM18*), rs17782313 (*MC4R*) for BMI and hip; rs17782313 (*MC4R*), rs7132908 (*FAIM2*) for waist and body shape. Non-standardized effect sizes were largest with rs1121980 (*FTO*) for all three traits (0.31*kg⋅m*^-2^, 0.70 *cm* and 0.47*cm*), but smallest with rs7647305 (*ETV5*) (0.08*kg⋅m*^-2^, 0.06*cm* and 0.09*cm*).

Based on the distribution of PRS and cumulative effects of the 12 variants on the three traits shown in Figure S1 (a2, b2, c2), we conclude that: 1) PRS was normally distributed, with comparable ranges of 0.08-1.75, 0.04-1.67 and 0.08-1.70 for the three traits. The majority (68.27%) of individuals () also showed similar patterns of PRS (0.90 ± 0.21, 0.84 ± 0.21 and 0.88 ± 0.20). 2) for each level of PRS, the distribution of the three traits are generally normal within 0.4-1.3 but skewed with < 0.4 or >1.3; 3) The means of the three traits increased linearly with PRS, with on average each additional unit associated with increments (*P*) of 1.73 *kg/m*^2^ (1.19×10^-27^), 4.08*cm* (5.20×10^-20^), 3.07*cm* (1.08×10^-19^), respectively.
